# Neurodevelopment among children exposed to HIV and uninfected in sub‐Saharan Africa

**DOI:** 10.1002/jia2.26159

**Published:** 2023-11-01

**Authors:** Michelle A. Bulterys, Irene Njuguna, Mary Mahy, Laurie A. Gulaid, Katheen M. Powis, Catherine J. Wedderburn, Grace John‐Stewart

**Affiliations:** ^1^ Department of Epidemiology University of Washington Seattle Washington USA; ^2^ Department of Global Health University of Washington Seattle Washington USA; ^3^ Kenyatta National Hospital Nairobi Kenya; ^4^ UNAIDS Geneva Switzerland; ^5^ UNICEF eastern and southern Africa Regional Office Nairobi Kenya; ^6^ Harvard Medical School Boston Massachusetts USA; ^7^ Department of Immunology and Infectious Diseases Harvard T.H. Chan School of Public Health Boston Massachusetts USA; ^8^ Department of Internal Medicine and Pediatrics Massachusetts General Hospital Boston Massachusetts USA; ^9^ Department of Pediatrics and Child Health and Neuroscience Institute University of Cape Town Cape Town South Africa; ^10^ Department of Clinical Research London School of Hygiene & Tropical Medicine London UK; ^11^ Department of Pediatrics University of Washington Seattle Washington USA; ^12^ School of Medicine University of Washington Seattle Washington USA

**Keywords:** CHEU, children who are HIV‐exposed uninfected, HEU, neurodevelopment, perinatal HIV exposure, sub‐Saharan Africa

## Abstract

**Introduction:**

The population of 16 million children exposed to HIV and uninfected (CHEU) under 15 years of age continues to expand rapidly, and the estimated prevalence of CHEU exceeds 20% in several countries in sub‐Saharan Africa with high HIV prevalence. Some evidence suggests that CHEU experience suboptimal neurodevelopmental outcomes compared to children born to women without HIV. In this commentary, we discuss the latest research on biologic and socio‐behavioural factors associated with neurodevelopmental outcomes among CHEU.

**Discussion:**

Some but not all studies have noted that CHEU are at risk of poorer neurodevelopment across multiple cognitive domains, most notably in language and motor skills, in diverse settings, ages and using varied assessment tools. Foetal HIV exposure can adversely influence infant immune function, structural brain integrity and growth trajectories. Foetal exposure to antiretrovirals may also influence outcomes. Moreover, general, non‐CHEU‐specific risk factors for poor neurodevelopment, such as preterm birth, food insecurity, growth faltering and household violence, are amplified among CHEU; addressing these factors will require multi‐factorial solutions. There is a need for rigorous harmonised approaches to identify children at the highest risk of delay. In high‐burden HIV settings, existing maternal child health programmes serving the general population could adopt structured early child development programmes that educate healthcare workers on CHEU‐specific risk factors and train them to conduct rapid neurodevelopmental screening tests. Community‐based interventions targeting parent knowledge of optimal caregiving practices have shown to be successful in improving neurodevelopmental outcomes in children and should be adapted for CHEU.

**Conclusions:**

CHEU in sub‐Saharan Africa have biologic and socio‐behavioural factors that may influence their neurodevelopment, brain maturation, immune system and overall health and wellbeing. Multidisciplinary research is needed to disentangle complex interactions between contributing factors. Common environmental and social risk factors for suboptimal neurodevelopment in the general population are disproportionately magnified within the CHEU population, and it is, therefore, important to draw on existing knowledge when considering the socio‐behavioural pathways through which HIV exposure could impact CHEU neurodevelopment. Approaches to identify children at greatest risk for poor outcomes and multisectoral interventions are needed to ensure optimal outcomes for CHEU in sub‐Saharan Africa.

## 1 INTRODUCTION

**Figure 1:**
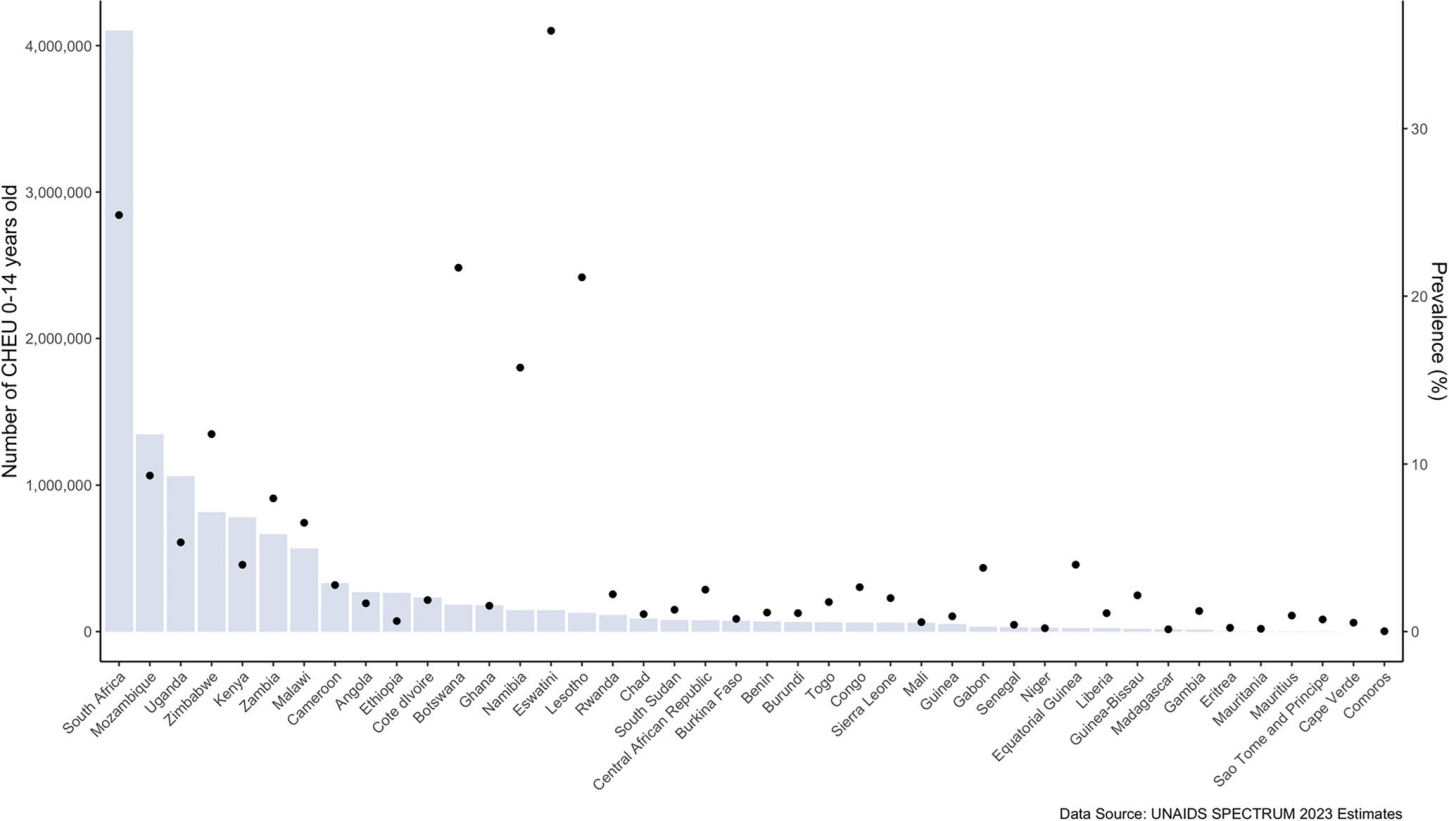
Double‐axis figure of the number of CHEU aged 0–14 years, and prevalence of CHEU, by sub‐Saharan African country (UNAIDS 2023 estimates). This double‐axis figure depicts the (x axis) number of children exposed to HIV and uninfected (CHEU), 0–14 years old, and the (y axis) prevalence of CHEU among the child population aged 0–14 years, by sub‐Saharan African country. Figure by Drs. Michelle Bulterys and Mary Mahy, using UNAIDS 2023 estimates.

**Figure 2:**
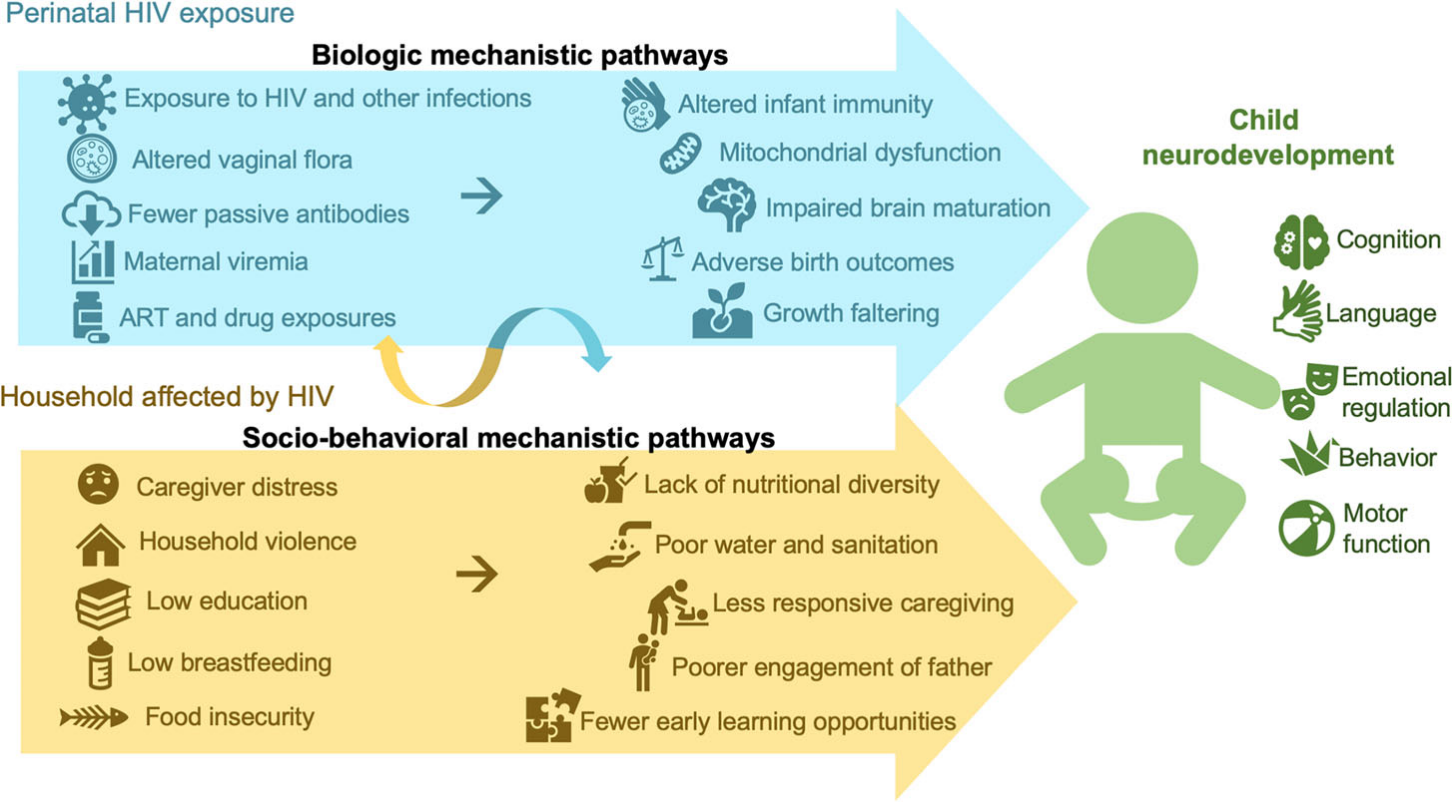
Biologic and socio‐behavioural mechanistic pathways through which CHEU status might impact neurodevelopment. This conceptual model summarises the potential biologic and socio‐behavioural mechanistic pathways through which perinatal HIV exposure might impact child neurodevelopment among children exposed to HIV and uninfected (CHEU).

Successful prevention of vertical infant HIV acquisition has resulted in an expanding population of 16 million children exposed to HIV and uninfected (CHEU) under 15 years of age, according to the 2023 UNAIDS Spectrum estimates [1, 2, 3]. CHEU represent over three‐quarters of all children born to the 1.3 million women living with HIV who give birth annually worldwide, the majority of whom reside in sub‐Saharan Africa [1, 4]. UNAIDS estimates the prevalence of CHEU among all children <15 years in the population exceeds 20% in several sub‐Saharan African countries, with over one million CHEU born every year (Figure 1) [1, 4]. CHEU are at higher risk of adverse health outcomes, including suboptimal neurodevelopment, compared to children who are HIV‐unexposed and uninfected (CHUU) [1, 4–17]. A recent meta‐analysis of eight high‐quality studies combining neurodevelopment data from ∼5000 CHEU and CHUU, primarily from sub‐Saharan Africa, found CHEU had significantly lower scores in expressive language and gross motor domains by age 2 years across a variety of settings and assessment tools [6, 11, 18–26]. Even subtle early neurodevelopment impairments can have lifelong physical and mental wellbeing implications. Early interventions in the first 1000 days of life, from conception through a child's second birthday, can greatly improve outcomes [27, 28, 29, 30, 31]. This commentary discusses potential biologic and socio‐behavioural mechanistic pathways that could synergistically impact neurodevelopment among CHEU and synthesises existing literature on the key ingredients for cultivating optimal neurodevelopment for CHEU (Figure 2).

## 2 DISCUSSION

### 2.1 *In‐utero* exposure to HIV and the intrauterine environment

Infant brain development *in utero* may be influenced by exposures to infectious pathogens or to drugs, and both may play a role in the context of maternal HIV, but these mechanistic pathways have yet to be fully established. High levels of HIV viremia among mothers during pregnancy have been associated with poorer expressive language and motor skills among CHEU [32]. HIV antigen and ribonucleic acid are detectable in placental and foetal membranes and it is possible that exposure to the HIV virus leads to inflammatory changes that influence neurodevelopment [33, 34]. The HIV virus can alter vaginal microbiota which, in turn, has also been associated with child neurodevelopmental delay [35]. Pregnant women with HIV exhibit six times higher incidence of endometrial, placental and amniotic infections, such as cytomegalovirus, which could impact neurodevelopment among CHEU [13, 36–38]. Maternal immune activation, immunosuppression, lower transfer of passively transferred antibodies and altered cell‐mediated immune function may play a role in modifying neurodevelopmental outcomes [10, 13, 16, 39–42]. Additionally, maternal folic acid and iron deficiency during pregnancy have been associated with neurodevelopment and may be altered by perinatal exposure to HIV, but have not been adequately studied among CHEU [43]. Moreover, CHEU may themselves have immunologic changes that affect their neurodevelopment that warrant exploration [10, 44–46].

### 2.2 Antiretroviral therapy exposure

There are inconsistent and limited data on the influence of antiretroviral therapy (ART) exposure on CHEU neurodevelopment [11, 18, 47, 48]. Foetal ART exposure has been associated with subtle but significantly reduced immune function through 8 years [49, 50]. However, studies were subject to substantial confounding, as CHEU with foetal ART exposure were likely systematically different from CHEU born without the use of maternal ART. Some but not all studies have found ART exposure to be associated with substantial ART drug levels, febrile seizures, mitochondrial dysfunction and neurologic disorders in CHEU [51, 52, 53, 54]. Although some studies reported that a longer duration of foetal exposure to atazanavir‐based regimens was associated with lower language scores at 12 months, other studies at 24 months found no notable differences [32, 55–57]. In Botswana, foetal exposure to efavirenz (EFV)‐based regimens was associated with neurodevelopmental deficits among 2‐year‐old CHEU, compared to non‐EFV‐based regimens [58]. It is also critical to understand the safety of evolving HIV treatments during pregnancy; the World Health Organisation (WHO)’s recommendation to transition all individuals living with HIV to dolutegravir (DTG)‐based regimens provided a unique opportunity to assess the impact of foetal DTG exposure on CHEU neurodevelopment [59]. A recent multi‐site prospective longitudinal cohort of Kenyan CHEU found that compared to foetal exposure to an EFV‐based maternal ART, a DTG‐based regimen was associated with better gross motor scores at 1 year (Bulterys et al., included in this supplement). To date, there are scant neurodevelopment data following foetal exposure to long‐acting ART. With any observational study, unmeasured confounding could bias results; but this collective body of literature suggests that maternal ART could play a role in CHEU neurodevelopment. Ultimately, the benefits of ART for maternal HIV treatment and prevention of vertical transmission greatly outweigh the risks of rare and modest adverse outcomes. However, employing rigorous scientific practices to identify the safest drugs for use in pregnancy and the postnatal period for women living with HIV remains important [57].

### 2.3 Brain maturation and imaging studies

Rapid brain growth in the first 1000 days of life is vital for developing healthy cognitive systems [60, 61]. A recent magnetic resonance imaging (MRI) study in South Africa observed that ART‐exposed newborns had significantly lower volumes of total grey matter and size of the caudate nucleus, a key component of the basal ganglia, than CHUU [60]. These structures are fundamental for brain function, and alterations have been associated with neurologic disorders (e.g. autism spectrum disorder, attention‐deficit disorders and schizophrenia) [61, 62, 63, 64]. This association was strongest among mothers with lower CD4 cell counts in pregnancy [60], indicating that more advanced HIV viremia and corresponding immune abnormalities may be driving some differences. Separately, another South African study found smaller basal ganglia nuclei in CHEU [65], most notably associated with maternal ART initiation during pregnancy compared to pre‐pregnancy and higher HIV viremia, suggesting that ART may be protective due to its effect on maternal immune health [65]. Other MRI studies have detected significantly altered metabolites in the basal ganglia, including choline (regulator of mood and intelligence) and creatine (regulator of energy production) among older CHEU [66, 67, 68], while diffusion tensor imaging has revealed altered white matter microstructural integrity (essential for visuospatial and memory cognition) among CHEU [69, 70]. Finally, a recent magnetic resonance spectroscopy study suggests perinatal HIV exposure to be associated with neurometabolic patterns indicative of neuroinflammation, which may increase the risk of neurodevelopmental delay [71]. It will be important to pair cutting‐edge neuroimaging research, leveraging scalable low‐field MRI technologies, with contextually appropriate neurodevelopment assessments to determine whether neuroimaging could represent the earliest signal of suboptimal neurodevelopmental outcomes for which interventions could be developed and tested.

### 2.4 Birth outcomes

CHEU are twice as likely as CHUU to be born preterm (<37 weeks gestational age) and have low birth weight (<2500 grams), both of which are associated with increased risk of poorer neurodevelopment [10, 13, 23, 45, 72–75]. In South Africa, preterm birth modified the relationship between perinatal HIV exposure and poorer neurodevelopment; preterm CHEU had five times higher odds of delay compared to preterm CHUU [23]. Similarly, in a Kenyan cohort of CHEU, preterm birth was significantly associated with poorer gross motor scores at 1 year (Bulterys et al., included in this supplement). Among pregnant Ugandan women living with HIV, an increased risk of preterm birth was associated with maternal weight gain of less than 0.1 kg per week during gestation, highlighting the importance of supporting maternal nutrition during pregnancy [76]. Research is needed to identify modifiable predictors of preterm birth among CHEU.

### 2.5 Growth and nutrition

CHEU have higher prevalence of childhood stunting, wasting and microcephaly than CHUU, and growth faltering could lie on the pathway between HIV exposure and neurodevelopmental delay [77]. It is unclear whether suboptimal growth outcomes are amplified among the CHEU population, or simply a result of higher frequencies of exposures like preterm birth among CHEU [78, 79, 80, 81, 82]. Exclusive, prolonged breastfeeding has been shown to improve child growth and neurodevelopmental outcomes [20, 83]. However, despite increased exclusive and longer breastfeeding durations in CHEU than CHUU in some South African and Kenyan settings, evidence has shown that CHEU remain at higher risk of undernutrition, poorer growth and infectious morbidity [77, 84]. Poor maternal nutrition while lactating, despite optimal breastfeeding practices, could be one potential mechanism that explains growth faltering among breastfed CHEU [77, 85]. Households affected by HIV in sub‐Saharan Africa face multifactorial health and social disparities, including greater food insecurity and lower access to safe water, sanitation and hygiene (WASH), compared to the general population [20, 86], which significantly predict poorer neurodevelopment [32, 46, 55, 87]. In the Zimbabwean SHINE Trial, CHEU randomised to a combined food supplementation and WASH intervention performed significantly better on neurodevelopmental assessments compared to the standard of care; however, children randomised to either food supplementation or WASH alone did not exhibit neurodevelopmental improvements, demonstrating the importance of multi‐factorial interventions [88, 89]. Vigilant growth monitoring and nutritional support should be prioritised for CHEU, particularly for those who were born prematurely.

### 2.6 Home environment and caregiving

Beyond the biologic pathways described above, universal risk factors for child neurodevelopment, such as poor caregiver mental health, violence and food insecurity in the household, are amplified among CHEU [90, 91, 92]. Women living with HIV experience disproportionately high rates of intimate partner violence and food insecurity which considerably threaten a child's neurodevelopment and academic performance [28, 29, 91–104]. These factors can impact a caregiver's ability to care responsively for their children, and likely serve as potential confounders or modifiers along the biological pathways described above [96, 97, 98]. Couples affected by HIV also experience relationship dissolution more often than couples in the general population, and pregnant women living with HIV cite often fear of abandonment to be their most prominent barrier to HIV status disclosure [100, 105–107]. On average, HIV serodifferent couples separate five times more often when the female is the one living with HIV compared to the male, and this association was also compounded by financial insecurity [105, 107]. Attributable to many of these social disparities, women living with HIV are at high risk of stress, anxiety, depression and suicidal ideation [29, 108], and studies have consistently found a strong relationship between a mother living with HIV, distress and poorer child neurodevelopment [109, 110, 111]. Both paternal absence and poor maternal mental health can reduce the quantity and quality of parent‐child interactions, which could impact a child's exposure to responsive caregiving and early learning opportunities [112]. Despite the critical impact of these caregiver factors, mental health, relationship counselling and violence‐reduction interventions are rarely implemented in low‐resource settings where the healthcare cadre are overtaxed [113]. Thus, caregiver conflict and distress could further contribute to suboptimal neurodevelopment among CHEU, which calls for caregiver‐targeted interventions.

### 2.7 Programmatic support for CHEU

Sub‐Saharan Africa has the highest prevalence in the world of children <5 years at risk of not reaching their developmental potential, and CHEU represent a substantial proportion of this population [114]. Achieving healthy neurodevelopment by age 5 requires culturally contextual, multi‐factorial approaches. To address biologic risk factors faced by CHEU, it is critical to optimise maternal health; newer ART regimens promote maternal health through improved safety, ART adherence and viral suppression. The SHINE Trial described above, which tested the effects of improved WASH and food supplementation on CHEU neurodevelopment, only found evidence of benefit in the combined intervention arm [88, 115, 116]. There is a need for further research to inform the development of cross‐cutting interventions and normative guidance to best support CHEU.

Not every CHEU will need additional support, so systematic screening is necessary. CHEU are not tracked systematically, and screening may be challenging to implement in already overburdened, resource‐limited settings. Incorporating monitoring within existing Maternal and Child Health programmes, in which many HIV‐oriented programmes are already housed may be feasible. Ideally, these existing programmes serving the general population could adopt structured neurodevelopment training programmes that educate healthcare workers on CHEU‐specific risk factors and train them to conduct rapid neurodevelopmental screening tests to identify children at the highest risk of delay. Accurate and rapid screening tools that can be delivered by healthcare workers as well as lay people in the community are needed. The three most common screening tests used in sub‐Saharan Africa are the Ages and Stages Questionnaire, Strengths and Difficulties Questionnaire and Ten Questions Questionnaire; all three have strong interobserver reliability and cover different age bands [117, 118, 119].

The WHO and UNICEF currently recommend countries rely on the Nurturing Care Framework to improve early child development [112]. This framework consists of five interdependent domains—health, nutrition, responsive caregiving, safety and early learning opportunities—which have shown to result in optimal neurodevelopment, if adequately provided by caregivers. Delivering this education to caregivers in the first 2 years of life, when CHEU families are actively engaged in Maternal and Child Health care, maximises benefits at a critical time [31, 120, 121]. A large cohort study of 10,500 CHEU, from 23 clinics across Kenya, estimated the incidence of loss‐to‐follow‐up (LTFU) from perinatal care was >20 per 100 child‐years [122]. In this study, LTFU was significantly lower among CHEU who received food supplementation compared to those who did not (Hazard Ratio = 0.58) [122]. Food supplementation may incentivise caregivers to remain engaged in care. LTFU was more common among CHEU who were orphaned, malnourished, stunted or wasted, from rural residence, or had >3 siblings, in studies in Kenya, Malawi and Ethiopia [122, 123, 124]. Routine neurodevelopmental screenings should coincide with routine paediatric care visits, when children are already receiving immunisations and growth monitoring, to reduce the burden on caregivers and healthcare workers [125]. Mothers attending HIV services could also receive brief materials at each perinatal visit about the importance of at‐home stimulation, responsive caregiving, maternal mental health, WASH and diverse nutrition (and if needed, food supplementation) [125].

Existing early child development programmes can be leveraged for CHEU; successful interventions for promoting neurodevelopment in the general population are likely to be effective among CHEU. Clinical trials have demonstrated effectiveness in improving neurodevelopmental outcomes in resource‐limited settings and this evidence base should be leveraged and adapted for CHEU. Large meta‐analyses of interventions in low‐and‐middle‐income countries found that providing parents with education about responsive interaction, at‐home stimulation, and providing a safe and healthy home environment, were more effective in improving neurodevelopmental outcomes than interventions that targeted nutrition, water and sanitation [31, 120, 126, 127]. Interventions to promote responsive caregiving and infant stimulation techniques, such as book‐sharing, [128] significantly improve parental knowledge, frequency and quality of interactions, and child neurodevelopment [31, 125, 128]. It is critical to identify the optimal delivery models of caregiver‐targeted education in sub‐Saharan Africa, which may be adapted for varied regional settings. Video‐based interventions are efficient for reducing healthcare worker burden; in clinics with technological capabilities, educational videos for mothers in waiting rooms could improve child neurodevelopmental outcomes [129, 130, 131, 132]. There is also a need to reach caregivers who are not engaged in clinical care, through non‐clinic, community‐based or home‐delivered interventions that leverage community health workers and mobile health innovations [133].

In cases of suspected neurodevelopmental disability (as opposed to just delay), healthcare workers need clear and effective referral pathways to connect children to specialists for comprehensive assessments and treatment. Fortunately, there are efficacious evidence‐based treatments for children with developmental disabilities in high HIV‐burden settings which could be adopted [121, 134, 135]. In settings where specialists are few and costly, simple, community‐based interventions can be designed to support families requiring additional support. Iterative input from key stakeholders like in‐country HIV and paediatric care decision‐makers, health providers and caregivers will be fundamental for designing, delivering and monitoring these programmes. Most importantly, rigorous research that generates local evidence will be critical to secure governmental buy‐in and financial support, both essential for the successful implementation and sustainability of such programmes for CHEU.

## 3 CONCLUSIONS

Biological and socio‐behavioural factors can collectively contribute to child development. CHEU are at disproportionate risk of biologic, social and household factors that may threaten their ability to achieve optimal maturation of their brain, immune system, and overall health and wellbeing. Multidisciplinary research is needed to disentangle the modifiable aspects of and complex interactions between potential contributing factors.

## COMPETING INTERESTS

The authors have no competing interests to disclose.

## AUTHORS’ CONTRIBUTIONS

The initial topic of this commentary was developed by MAB and GJ‐S. MAB led manuscript development with detailed guidance and iterative feedback from IN, MM, LAG, KMP, CJW and GJ‐S. All authors reviewed the manuscript and approved it for publication.
